# Regional Bioelectrical Phase Angle Is More Informative than Whole-Body Phase Angle for Monitoring Neuromuscular Performance: A Pilot Study in Elite Young Soccer Players

**DOI:** 10.3390/sports10050066

**Published:** 2022-04-22

**Authors:** Tindaro Bongiovanni, Alessio Rossi, Athos Trecroci, Giulia Martera, F. Marcello Iaia, Giampietro Alberti, Giulio Pasta, Mathieu Lacome

**Affiliations:** 1 Performance and Analytics Department, Parma Calcio 1913, 43121 Parma, Italy; tindaro.bongiovanni@gmail.com (T.B.); mathlacome@gmail.com (M.L.); 2 Department of Computer Science, University of Pisa, 56126 Pisa, Italy; alessio.rossi2@gmail.com; 3 Department of Biomedical Sciences for Health, Università degli Studi di Milano, 20129 Milano, Italy; giampietro.alberti@unimi.it (F.M.I.); giumartera@gmail.com (G.A.); 4 Nutrition Department, Spezia Calcio, 19121 La Spezia, Italy; marcello.iaia@unimi.it; 5 Medical Department, Parma Calcio 1913, 43121 Parma, Italy; ghitopasta@hotmail.com; 6 Laboratory Sport, French Institute of Sport (INSEP), Expertise and Performance (EA 7370), 75012 Paris, France

**Keywords:** soccer, youth sport, body composition

## Abstract

Background: The objective of this study was to investigate the association between regional and total phase angle (PhA) with lower-body neuromuscular performance in young elite soccer players. Methods: Sixteen elite male soccer players (14.3 ± 1.0 years) participated in this study. Lower (LPhA)- and upper (UPhA)-hemisome PhA together with whole-body PhA (WBPhA) were measured by a bioelectrical-impedance analysis (BIA), while appendicular arm and leg lean soft tissue (ALST and LLST, respectively) were estimated. Urine osmolarity (UOsm) and urine-specific gravity (USG) were also considered. Sprints over 10 m and 20 m and countermovement jump (CMJ) tests were employed to evaluate neuromuscular performance. Results: LPhA (*p* = 0.003) and UOsm (*p* = 0.012) explained 62% of the variance in the 10 m sprint. UOsm (*p* = 0.001) and both LPhA (*p* < 0.001) and WBPhA (*p* = 0.024) explained 81% of the total variance in the 20 m sprint. The CMJ height was affected by LPhA (*p* < 0.001) and UOsm (*p* = 0.024), which overall explained 68% of its variance (*p* < 0.05), while 93% of the CMJ power variance was explained by LPhA (*p* < 0.001), ALST (*p* < 0.001), and WBPhA (*p* = 0.011). Conclusions: Regional PhA is a relevant and non-invasive tool to monitor lower-body neuromuscular performance in elite youth soccer. Specifically, LPhA may be favored over WBPhA as more informative.

## 1. Introduction

Estimation of body composition is a cornerstone of human nutrition assessment for clinical researchers, physicians, and nutritionists. In sports, assessing body composition during the competitive season provides valuable information that can help sports professionals to assess the efficacy of the training process and also to monitor the athletes’ health status [1,2,3]. Several studies have reported that a reduction in body fatness and an increase in lean mass improves endurance, agility, and power performance in athletes [4,5,6].

An accurate estimate of athletes’ body composition may vary in accordance with the different assessment techniques used. Even if laboratory testing methods are the most accurate, they are time consuming, expensive and might expose athletes to unnecessary radiation, albeit being harmless [7], which make their day-to-day use unfeasible [7]. Conversely, even if field-based methods or equipment to assess body composition presents less accuracy, they are accessible, easy to use, minimally invasive, and cost effective (e.g., bioelectrical-impedance analysis, BIA) with a more feasible day-to-day use compared with laboratory methods [2]. Among these techniques, BIA represents a more affordable and convenient method of estimating body composition by measuring raw parameters such as resistance, reactance, and phase angle (PhA). This technique involves the application of a weak alternating current throughout the body, with surface-contact electrodes placed on the foot and hand. The electrical conduction in the body is related to the water and electrolyte distribution [8]. Besides the raw bioelectrical parameters, PhA has been suggested to be a biomarker of cellular health and cell-membrane integrity [9] and descriptive of the intracellular/extracellular water ratio [10,11]. Thanks to these features, BIA-based assessment of PhA has been widely used among both the general population and athletes [12] in recent years. In sports and exercise science, studies have recently focused their attention on PhA by exploring its association with health and physical performance in soccer. For example, Martins et al. [13] reported that young soccer players with higher whole-body PhA values had better performance in sprinting speed and repeated-sprint ability. Likewise, a recent study reported a significant correlation between whole-body PhA (WBPhA) and vertical-jump (positive correlation) and sprint performance (inverse correlation) in youth soccer [14]. Moreover, Nabuco et al. [15] showed that WBPhA was inversely associated with perceived fatigue and positively related with sprint performance in soccer players.

Hetherington-Rauth [16] reported that PhA may have the potential to be used as a marker of functional muscle mass, which is important when it comes to assessing handgrip strength and countermovement jump power of athletes from different sports [16]. In this view, Obayashi et al. [17] showed that WBPhA strongly correlated with muscle performance (e.g., muscle strength and power). Pollastri et al. [18] showed a positive association between whole-body PhA and mean power output of short-duration effort (e.g., 15 s) in elite adult cyclists. Although the type of participants and practiced sport were divergent (adolescent vs. adult and soccer vs. cycling), these results provide primary evidence on the role of PhA as a determinant of physical performance in adolescent and adult athletes.

In contrast to the commonly employed WBPhA analysis, the assessment of regional or segmental body composition adds specificity to the body-composition profile (e.g., arm- and leg-fat mass, and upper- and lower-body lean-soft-tissue mass) and could be potentially more informative than the whole body itself [19]. Actually, measuring raw BIA parameters of different body segments overthrows the theoretical limits underpinning conventional BIA analysis (i.e., considering the human body as the set of five cylinders of constant resistivity) [20]. A recent application of this regional approach can be found in a previous pilot study [21] in which the authors showed a positive association between lower-hemisome PhA (LPhA) and vertical-jump performance in male elite soccer players. Further, a positive relation between hand-grip strength and upper-hemisome PhA (UPhA) was reported in female volleyball players [22]. Additionally, Marra et al. [23] reported that, while the WBPhA did not change, the LPhA decreased during a three-week stage race in professional male cyclists, probably mirroring cellular damage and/or a change in cell number following the competitive event.

Of note, these preliminary studies shed a light on the potential use of regional PhA (i.e., LPhA and UPhA) for monitoring neuromuscular performance, which may be more informative than WBPhA, especially in soccer where technical (e.g., kicking) and physical (e.g., sprinting and jumping) demands mainly involve a specific body region. Moreover, additional information is needed to improve the practitioners’ understanding on the association between body-region data and physical performance in a young population of players (e.g., under 14 years of age) for whom characteristics may undergo an ongoing change during the season.

Therefore, the aim of this study was to examine the association between regional (i.e., UPhA and LPhA) and total (i.e., WBPhA) phase angle with sprint and jump performance in soccer players. Since the lower body is notably involved in sprinting and jumping, we hypothesized that LPhA would be more informative than WBPhA by exhibiting a better association.

## 2. Materials and Methods

### 2.1. Study Design

**Figure 1:**
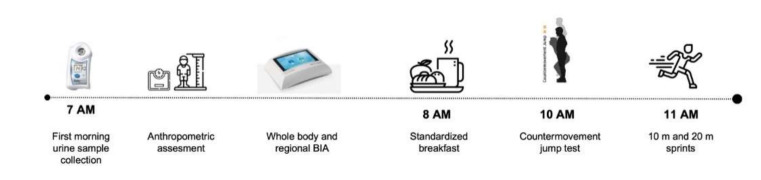
A schematic representation of the testing protocol. Note: BIA = bioelectrical-impedance analysis.

An observational study design was adopted to assess the contribution of whole-body and regional raw bioelectrical BIA parameters on performance in a group of U14 elite soccer players. The assessments were performed during the in-season phase (April) of a competitive championship. Athletes underwent whole-body and regional BIA analysis in a fasted state, then a standardized breakfast that was similar in macronutrient distribution for all athletes (65%, 20%, 15%, carbohydrates, fat and protein, respectively) was consumed [24]. After 2 h, athletes were tested (Figure 1). All players were requested to abstain from using dietary supplements, from drinking caffeinated drinks, and from exercising at moderate-to-high intensity (except during the tests included in the experimental design) before (within 48 h) and the day of the study.

### 2.2. Participants

Sixteen male elite soccer players (ages 14.3 ± 1.0 years, body weight 63.2 ± 6.8 kg, height 176.0 ± 5.7 cm, fat mass 11.2 ± 2.16%), from the same professional club competing in the Italian first division voluntarily participated in the study. The study was performed during the in-season phase to ensure the participants were optimally accustomed to training. Inclusion criteria were: (i) practicing soccer at an elite level for at least 4 years, (ii) having obtained sports eligibility, (iii) weekly training routine of at least 3 sessions and 1 played match, (iv) being Caucasian to keep the resistance index (height^2^/resistance) as homogeneous as possible [25]. Exclusion criteria were: (i) presence of severe lower-limb injuries from the last year to 1 month prior to the experimental study, and (ii) recent history of febrile illness. After being fully informed about the experimental procedures, aims, and potential risks of the study, all participants and their parents provided a written consent before starting the study. In accordance with the Declaration of Helsinki [26], the study was approved by the Ethics Committee of the local University.

### 2.3. Bioimpedance and Body Composition Analysis

Prior to examining the bioimpedance and body composition, height and body mass were recorded to the nearest 0.1 cm and 0.1 kg with a standing stadiometer (model Seca 217, Seca AG, Basel, Switzerland) and a high-precision mechanical scale (model Seca 877, Seca AG, Basel, Switzerland), respectively. Whole-body and regional bioimpedance parameters of resistance, reactance and PhA were obtained using a phase-sensitive bioelectrical analyzer (BIA 101 BIVA PRO, Akern Bioresearch, Florence, Italy). The device emits an alternating sinusoidal electric current of 250 μA at an operating monofrequency of 50 kHz (±0.1%). The device was calibrated using the standard control circuit that has a known impedance, supplied by the manufacturer. The test–retest reliability was previously reported as expressed [21,27] by the coefficient of variation, which was 0.3%, 0.8% and 0.9% for resistance, reactance and PhA, respectively.

**Figure 2:**
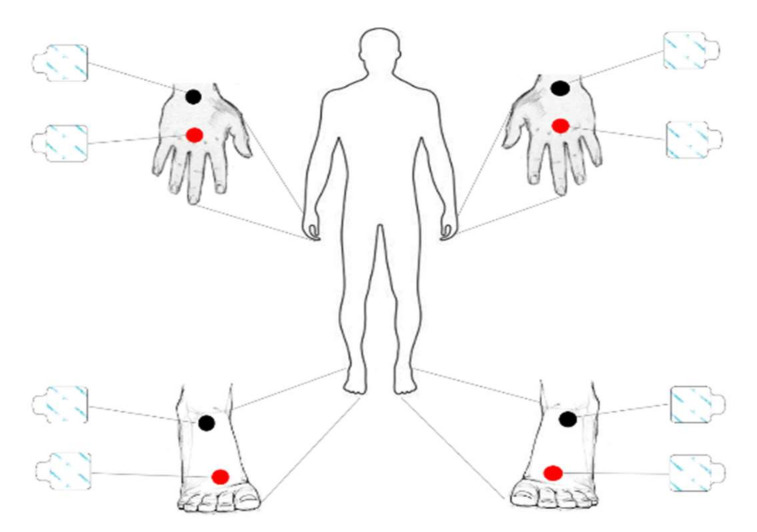
Scheme of the electrodes positioning. Black colors identify the sensing electrodes (proximal) while red colors identify the injection electrodes (distal).

Participants were positioned supine with a leg opening of 45° with respect to the midline of the body, and with the upper limbs positioned 30° away from the trunk (Figure 2). After cleaning the skin with alcohol pads, four adhesive electrodes (Biatrodes, Akern Bioresearch, Florence, Italy) were placed on the back of the hands and other four electrodes on the neck of the corresponding feet, keeping a distance of 5 cm between each electrode [27]. The proximal hand electrode was positioned between the radial and ulnar styloid processes, directly superficial to the distal radioulnar joint. The distal hand electrode was positioned in the center of the third, proximal phalanx. The proximal foot electrode was placed directly between the medial and lateral malleoli at the ankle. The distal foot electrode was placed immediately proximal to the second and third metatarsophalangeal joints.

Fat-free mass (FFM), fat mass (FM), percentage of fat mass (FM%) and appendicular arm and leg lean soft tissue (ALST, LLST) were estimated using specific equations developed for the athlete population [28,29].

### 2.4. Urine

Participants were instructed to wake up before 7 AM, void their bladder and collect their first morning urine sample in 100 mL sterile, individual, clear plastic containers to fill a minimum of 50 mL. For each returned sample, urine-specific gravity (USG) and urine osmolarity (UOsm) were measured with a hand-held refractometer (Atago, Tokyo, Japan) and osmometer (Osmocheck, Vitech Scientific, Horsham, UK), respectively. The effect of hydration status could affect the players performance [30,31,32,33]. For this reason, we decided to also record the urine samples in order to control the mediation effect of hydration status on the relationship between body composition and players’ performance. Various biomarkers of urine concentration were investigated to monitor individual daily hydration levels. Specifically, UOsm is the most precise, non-invasive biomarker available to evaluate the 24 h hydration process, as it represents the net sum of water gains, losses and neuroendocrine responses that act to maintain body-water homeostasis and rapidly respond to changes in daily water intake [34,35]. One alternative method for measuring urine concentration with greater clinical and field applicability is USG, which can be easily measured by clinicians. An acceptable euhydration cutoff has been previously reported as <700 to <830 mOsm/kg for UOsm [35,36] and <1.020 g/cm^3^ for USG [36]. Armstrong and colleagues found the measures of UOsm to be used interchangeably with USG, suggesting this as another potential marker [37].

### 2.5. Lower-Limb Neuromuscular Performance

All players followed a standardized warm-up prior to undergoing the field-based testing battery including vertical jump and sprint assessments. The sprinting tests were conducted outdoors on an artificial turf, while the vertical jump test was performed inside a gym. The two tests were separated by 10 min of rest.

*10 m and 20 m sprint performance.* Running time for 10 m- and 20 m-sprint performance was obtained by an electronic timing-gate system (Witty, Microgate, Bolzano, Italy) with the gates fixed at 0.7 m above the ground at the starting (0 m), middle distance (10 m) and finish lines (20 m). To prevent an early triggering of the beam on departure, the players were asked to start 0.3 m back from the timing gates. At their volition, the participants started accelerating maximally up to 20 m. There were allowed three trials with a 2 min recovery in between [38]. The best time recorded for 10 m and 20 m were considered for the analysis.

*Countermovement jump (CMJ)*. The Optojump next system (Optojump Next System, Microgate, Bolzano, Italy) was used to indirectly record vertical-jump height for each participant. During the trial, the participants were asked to jump keeping their hands on their hips and without bending the legs from takeoff and landing phase. Before being tested, each participant was allowed to perform a practice jump. Then, the participants performed three CMJ trials interspersed by 2 min of recovery, and the best performance was used for the analysis. Besides jump height, jump power calculation was also included by the formula of Lewis et al. [39] as shown in Equation (1).

power = √4.9 × weight × √(jump height) × 9.81 (1)

Weight and jump height were provided in kg and cm, respectively.

### 2.6. Statistical Analysis

The assumption of normality was checked by the Shapiro–Wilk test. All descriptive statistics of anthropometric characteristics, total and regional BIA parameters, urine-sample parameters, and lower-limb neuromuscular performance tests are provided as mean and standard deviation. A Pearson’s correlation analysis was conducted in order to assess the relationships among individuals’ characteristics (i.e., total and regional BIA parameters, and urine-sample parameters) and players’ performances. Additionally, based on the correlation outputs, a stepwise linear-regression analysis was performed in order to assess the multidimensional influence of individuals’ characteristics on the players’ performance. In particular, a forward-selection approach was used in this study. The regression model was first created with no variables, then the independent features were added in the regression model one at a time from the most to least correlated. If the regression model significantly improved its goodness of fit by adding an independent feature, then it was maintained in the model, otherwise it was removed. This process was repeated until no significant improvements were found. The dependent features (performance) used in this study were 10 m and 20 m sprint, jump height and power during CMJ. The independent features included in this analysis were LPhA, UPhA, WBPhA, ALST, LLST, FM, FFM, BMI, USG, and UOsm. All the analyses were performed by using Python 3.8 language programming. An alpha threshold of 5% was set to identify statistical significance (*p* < 0.05).

## 3. Results

**Figure 3:**
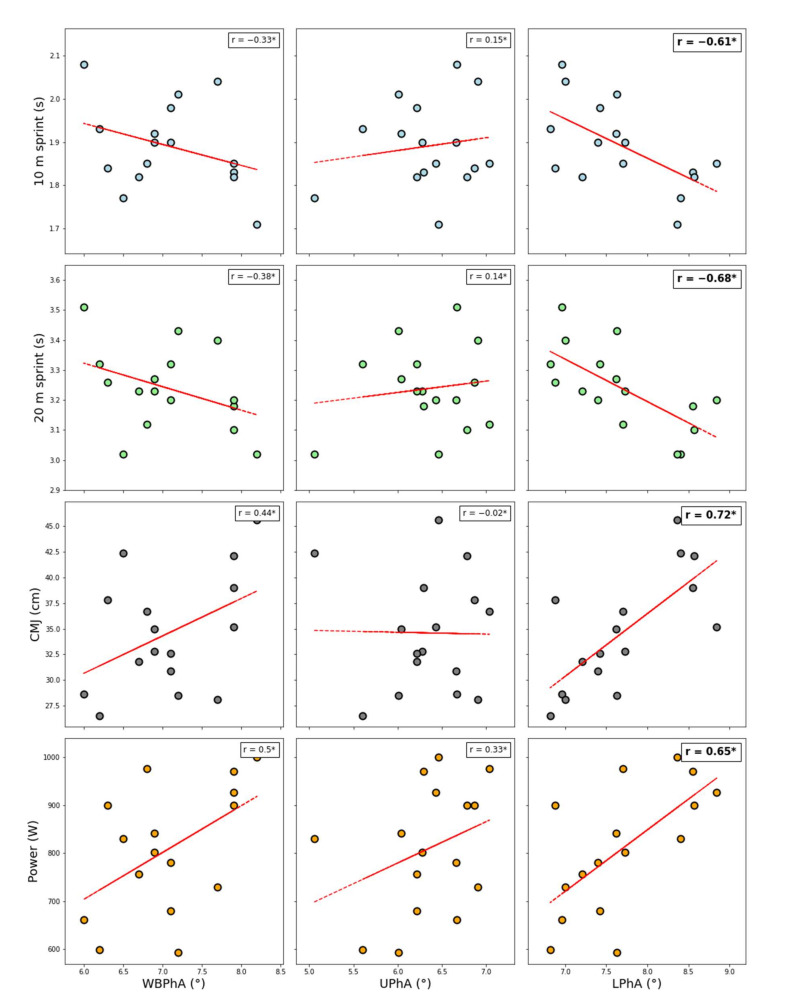
Correlation analysis between lower-body neuromuscular performance (CMJ height and power, 10 m and 20 m sprint) and BIA parameters (WBPhA, UPhA, LPhA). Note: CMJ = countermovement jump, WBPhA = whole-body phase angle, UPhA = upper-hemisome phase angle, LPhA = lower-hemisome phase angle. * Significant (*p* < 0.05) correlation.

The descriptive statistics expressed as mean and standard deviation of anthropometric characteristics, whole-body and regional BIA parameters, urine samples, and soccer-specific performance tests are shown in Table 1. Figure 3 provides the correlation analysis between whole-body and regional phase-angle parameters and players’ performances (i.e., 10 m and 20 m sprint, CMJ and power). LPhA showed a stronger correlation with players’ performance compared to UPhA and WBPhA (Figure 3) indicating that the phase angle (PhA) of lower limbs is more related to sprinting and power performance compared to the PhA of the upper limbs and whole body. Additionally, urine parameters (i.e., Uosm and Usg) showed a moderate negative correlation with 10 m sprint (r = −0.48 and r = −0.47, respectively) and 20 m sprint (r = −0.47 and r = −0.46, respectively), while a low-to-moderate positive correlation was shown with CMJ (r = 0.38 and r = 0.36, respectively) and power (r = 0.35 and r = 0.34, respectively). Table 2 shows the regression models created by the stepwise approach. A total of 62% of the variance for 10 m sprint is explained by LPhA and UOsm. UOsm and both lower-limb and whole-body PhAs affected the 20 m sprint, explaining 81% of the total variance. The CMJ height was correlated with LPhA and UOsm (r^2^ = 0.68, *p* < 0.001), while 93% of the power variance was explained by ALST, LPhA and WBPhA. LPhA was the only BIA parameter linked to all players’ performance.

## 4. Discussion

The purpose of this study was to examine the association of regional (i.e., UPhA and LPhA) and total (i.e., WBPhA) phase angle with sprint and jump performance in young elite soccer players. The main findings were: (1) LPhA showed higher correlation with lower-body neuromuscular performance than WBPhA and UPhA (Figure 3); (2) more than 80% of the variance in the 20 m-sprint performance and 93% of the variance in the CMJ performance were explained using only BIA-related measures and hydration-status biomarkers; (3) LPhA was the only parameter linked to all lower-body neuromuscular performance. These findings are in line with our previous hypothesis that regional PhA (i.e., LPhA) would be more informative than the whole-body measure for monitoring lower-body neuromuscular performance. This also suggests that regional BIA could be an interesting tool in the sports-scientist toolbox to indirectly monitor non-invasively lower-body neuromuscular status in season.

Soccer is an intermittent sport consisting of acyclical and unpredictable changes in activity, involving accelerations, decelerations, changes of direction, tackles, and jumps, as well as other intense actions such as kicking and dribbling [40,41], and is characterized by the engagement of both the lower and upper limbs. Indeed, the regional bioelectrical parameters could offer potential predictive markers of performance. As hypothesized, LPhA showed a higher correlation with 10 m- and 20 m-sprint and jump performance than UPhA and WBPhA (r = 0.65 to 0.72 vs. 0.33 to 0.50). To the best of our knowledge, this is the first study to investigate the association between regional PhA and performance in elite youth soccer, regardless of the mostly used indirect parameters of body composition such as FFM and FM. While one study did not report any improvement when using regional measures of PhA to estimate lower-body power compared with WBPhA [16], regional BIA approaches have been proven effective in individual and team sports to monitor health, training-induced changes, and fitness level [23,42]. This is in accordance with the results of a previous study [21] showing the potential superior usefulness of LPhA, compared with WBPhA, as a surrogate marker of lower-body neuromuscular performance in elite soccer players.

The multiple-regression analysis (Table 2) indicates that bioimpedance and body-composition assessment can provide useful information to better monitor sprint and jump performance. Notably, BIA-related parameters were among those explaining 62% (i.e., LPhA) and 81% (i.e., WBPhA and LPhA) of the variance in the 10 and 20 m sprint, respectively. In CMJ, LPhA helped to explain 68% and 93% of the variance in the jump height and power, respectively. A large proportion of vertical jump- and sprint-performance variance can then be estimated solely from LPhA-related measures. Of note, this finding is in line with that observed by Bongiovanni and colleagues [21] in adult professional soccer players. The authors revealed that changes in LPhA were strongly related with changes in jump performance, explaining ~61% of the total variance. LPhA is an affordable and convenient tool that allows practitioners to quickly and non-invasively collect lower-body neuromuscular-related information from all players [27]. Given that CMJ and sprinting tasks require players to undergo maximal neuromuscular efforts that could represent an additional load for players, they remain a practical challenge to implement in soccer clubs [39]. In a real-world setting, especially when schedules start to be congested, the time available as well as players and staff buy-in for testing is limited. Thus, we believe that regional BIA approaches (non-invasive and easy to perform) offer practitioners a very effective strategy to obtain field-based information on players’ lower-body neuromuscular readiness. Practitioners and coaching staff may benefit from such information as they could decide to retrieve data on their players’ lower-body readiness by LPhA at any time during the competitive season, without taxing the athletes’ body.

The present study has limitations that should be acknowledged. First, this study used a cross-sectional design that does not allow establishing a direct cause–effect relationship. Longitudinal studies are warranted to help determine an evolutionary character of LPhA on neuromuscular performance over time in the attempt to clarify its role within the monitoring process. Second, while we observed strong relationships between PhA and lower-body neuromuscular performance, our sample size was relatively small. Future studies will have to employ a larger sample size (perhaps considering more age categories) in order to corroborate our preliminary findings or not.

## 5. Conclusions

This study showed that monitoring regional PhA is more informative than total PhA on sprint and vertical-jump performance in elite young soccer players. These results confirm the promising use of regional LPhA to monitor regularly, non-invasively and effectively neuromuscular performance of lower-body segments, which are physically taxed in soccer.

## Figures and Tables

**Table 1:** The anthropometric characteristics, whole-body and regional BIA parameters, urine samples, and soccer-specific performance tests of the participants.

Characteristics	Feature	Mean	SD	95% CI
Anthropometry	age (years)	14.3	1.2	[11.95, 16.65]
	Body weight (kg)	63.2	6.8	[49.87, 76.53]
	Height (cm)	176.0	5.7	[164.83, 187.17]
	BMI	20.4	1.4	[17.66, 23.14]
BIA	Resistance	518.26	45.96	[428.18, 608.34]
	Reactance	64.26	6.74	[51.05, 77.47]
	WBPhA (°)	7.08	0.68	[5.75, 8.41]
	Upper-hemisome resistance (Ohm)	527.79	43.69	[442.16, 613.42]
	Upper-hemisome reactance (Ohm)	58.62	5.73	[47.39, 69.85]
	UPhA (°)	6.34	0.51	[5.34, 7.34]
	Lower-hemisome resistance (Ohm)	456.72	45.35	[367.83, 545.61]
	Lower-hemisome reactance (Ohm)	61.12	6.35	[48.67, 73.57]
	LPhA (°)	7.69	0.67	[6.38, 9]
	FFM (Kg)	56.11	5.50	[45.33, 66.89]
	FM (Kg)	7.13	1.85	[3.5, 10.76]
	FFM (%)	88.83	2.16	[84.6, 93.06]
	FM (%)	11.17	2.16	[6.94, 15.4]
	ALST (Kg)	5.09	0.77	[3.58, 6.6]
	LLST(Kg)	16.25	1.81	[12.7, 19.8]
Urine	UOsm (mOsm/KgH_2_O)	856.88	226.24	[413.45, 1300.31]
	USG (g/cm^3^)	1.019	0.007	[1.01, 1.03]
Performance variables	10 m sprint (s)	1.89	0.10	[1.69, 2.09]
	20 m sprint (s)	3.24	0.14	[2.97, 3.51]
	CMJ (cm)	34.6	5.7	[23.43, 45.77]
	Power (W)	808.9	132.0	[550.18, 1,067.62]

Note: SD = standard deviation, 95% CI = 95% confidence intervals, BMI = body-mass index, WBPhA = whole-body phase angle, UPhA = upper-hemisome phase angle, LPhA = lower-hemisome phase angle, FFM = fat-free mass, FM = fat mass, ALST = appendicular arm lean soft tissue, LLST = appendicular leg lean soft tissue, UOsm = urine osmolarity, USG = urine-specific gravity, CMJ = countermovement jump.

**Table 2:** Stepwise regression analysis results.

Variables	Parameters	Coef [95% CI]	SD	t	*p*-Value	r^2^
10 m sprint	const	2.80 [2.34, 3.26]	0.21	13.27	<0.001	0.62
	LPhA	−0.09 [−0.15, −0.04]	0.03	−3.64	0.003	
	UOsm	−2.27 × 10^−4^ [−8.35 × 10^−4^, −5.67 × 10^−5^]	7.57 × 10^−5^	−2.91	0.012	
20 m sprint	const	4.52 [4.05, 4.99]	0.22	21.02	<0.001	0.81
	LPhA	−0.21 [−0.30, −0.13]	0.04	−5.75	<0.001	
	UOsm	4.11 × 10^−4^ [1.13 × 10−4, 1.51 × 10^−5^]	8.16 × 10^−5^	−4.65	0.001	
	WBPhA	0.10 [0.02, 0.18]	0.04	2.58	0.024	
CMJ height	const	−21.97 [−45.72, 1.78]	10.99	−2.00	0.05	0.68
	LPhA	6.23 [3.33, 9.13]	1.34	4.64	<0.001	
	UOsm	0.01 [0.002, 0.02]	0.00	2.56	0.024	
CMJ power	const	−311.88 [−585.99, −37.77]	125.81	−2.48	0.029	0.93
	ALST	146.12 [110.97, 118.27]	16.13	9.06	<0.001	
	LPhA	113.78 [66.97, 160.60]	21.49	5.30	<0.001	
	WBPhA	−70.44 [−121.45, −19.44]	23.41	−3.01	0.011	

Note: SD = standard deviation, 95% CI = 95% confidence intervals, t = statistics score, r^2^ = coefficient of determination, CMJ = countermovement jump, WBPhA = whole-body phase angle, UPhA = upper-hemisome phase angle, LPhA = lower-hemisome phase angle, UOsm = urine osmolarity, ALST = appendicular arm lean soft tissue.

## Data Availability

The data are not publicly available due to privacy.
